# The dual effect of endoplasmic reticulum stress in digestive system tumors and intervention of Chinese botanical drug extracts: a review

**DOI:** 10.3389/fphar.2024.1339146

**Published:** 2024-02-21

**Authors:** Jinlong Zhang, Yanyu Chen, Bo Chen, Dajuan Sun, Zhen Sun, Junwei Liang, Jing Liang, Xin Xiong, Hua Yan

**Affiliations:** ^1^ Shandong University of Traditional Chinese Medicine, Jinan, Shandong, China; ^2^ Beijing University of Chinese Medicine, Beijing, China; ^3^ Affiliated Hospital of Shandong University of Traditional Chinese Medicine, Jinan, Shandong, China

**Keywords:** endoplasmic reticulum stress, unfolded protein response, digestive system tumor, Chinese botanical drug, dual effect

## Abstract

Endoplasmic reticulum (ER) homeostasis is essential for maintaining human health, and once imbalanced, it will trigger endoplasmic reticulum stress (ERS), which participates in the development of digestive system tumors and other diseases. ERS has dual effect on tumor cells, activating adaptive responses to promote survival or inducing apoptotic pathways to accelerate cell death of the tumor. Recent studies have demonstrated that Chinese botanical drug extracts can affect the tumor process of the digestive system by regulating ERS and exert anticancer effects. This article summarizes the dual effect of ERS in the process of digestive system tumors and the intervention of Chinese botanical drug extracts in recent years, as reference for the combined treatment of digestive system tumors with Chinese and modern medicine.

## 1 Introduction

Digestive system tumors are a general term for esophageal cancer (EC), gastric cancer (GC), hepatocellular carcinoma (HCC), colorectal cancer (CRC) and other cancers, which rank 10th, sixth, seventh, and fifth/eighth in global tumor incidence, respectively; 3 of the top 5 tumors in terms of global mortality rate belong to digestive system, posing a serious threat to human life and health (Cao W et al., 2021; Sung H et al., 2021). Due to the lack of early signs and symptoms, digestive system tumors are often difficult to be diagnosed in time, which makes them more challenging to be treated compared with other types of tumors as well (Matsuoka T and Yashiro M, 2020).

As the largest organelle in eukaryotic cells, the endoplasmic reticulum (ER) is involved in the synthesis of more than one-third of human body’s functional proteins and is closely linked to a wide range of life activities and disease processes (Oakes and Papa, 2015; Lemmer IL et al., 2021; Perkins and Allan, 2021). Under normal conditions, the protein quality control system is able to maintain the stability of proteases such as ATPases and glucose-regulated protein (GRP/BIP). Endoplasmic reticulum stress (ERS) occurs when the intracellular environment is altered such as an increase in ER unfolded proteins/misfolded proteins or an imbalance in Ca^2+^ homeostasis due to various causes. ERS is always activated in many digestive system tumors, correlates with the malignant biological behaviors of a wide range of tumor cells and plays a vital important role in the development of tumors (Madden E et al., 2019; Chen X and Cubillos-Ruiz JR, 2021): due to rapid growth of tumors, externally, tumor cells are susceptible to exposure to a local microenvironment of hypoxia, vascular insufficiency and nutrient deprivation; internally, tumor cells are genetically unstable yet have an increasing demand for protein synthesis, which together result in the chaos in protein synthesis and necessity for tumor cells to rely on ERS adaptation to survive early on, accelerating tumor growth. However, when ERS persists too long and severely, it can in turn induce apoptosis and autophagy in tumor cells preventing the diseases getting worse. Therefore, inhibition or enhancement of the ERS pathway in tumor cells through drug targeting correctly in different phases can inhibit the malignant biological behaviors of tumor cells, which has a very positive impact on cancer treatment and prognosis.

More and more research have shown that numerous extracts derived from widely used Chinese botanical drugs are able to intervene the development of digestive system tumors by regulating the ERS process, throwing new light on the treatment of these tumors (Liu R et al., 2020; Peng MF et al., 2021; Ma CX et al., 2023).

## 2 Mechanism of ERS-UPR

In response to ERS, cells initiate unfolded protein response (UPR) to promote protein folding, to degrade abnormal proteins and ultimately to return to ER homeostasis, which mainly consists of 3 parallel pathways mediated by protein kinase RNA-like ER kinase (PERK), inositol requiring enzyme1 (IRE1) and activating transcription factor 6 (ATF6) (Cao SS et al., 2016). In the physiological state, the molecular chaperone GRP78 binds to PERK, IRE1, and ATF6 and is in an unactive state; whereas, when ERS occurs, GRP78 actively dissociates from these three transmembrane sensing proteins to bind unfolded or misfolded proteins aggregated in the ER lumen, and the PERK, IRE1, and ATF6 pathways are consequently activated (Almanza et al., 2019; Gorbatyuk MS et al., 2020; Hetz C et al., 2020).

### 2.1 PERK signaling pathway

PERK, a type I ER-resident transmembrane protein, has dual enzymatic activity of serine/threonine protein kinase and endonuclease. When the unfolded/misfolded protein exceeds a certain amount, GRP78 detaches and binds to them, causing PERK to undergo autophosphorylation and then be activated to become p-PERK (Wang P et al., 2018). p-PERK specifically induces translation eukaryotic translation initiation factor 2α (eIF2α) phosphorylation of serine at position 51, which deprives eIF2α of its ability to initiate protein translation and reliefs ER burden (Oyadomari S et al., 2008; Rozpedek W et al., 2016). Meanwhile, p-eIF2α selectively activates the translation of ATF4 mRNA and upregulates ATF4 expression, which in turn promotes expression of CCAAT/enhancer binding protein homologous protein (CHOP) and growth arrest and DNA damage-inducible protein (GADD34), inhibiting B-cell lymphoma-2 (Bcl-2) transcription and ultimately inducing apoptosis (Hu H et al., 2019).

In the UPR signaling system, the PERK-eIF2α-ATF4 signaling pathway is the main pathway to induce CHOP expression (Liu Z et al., 2016).

### 2.2 IRE1 signaling pathway

Including IRE1α (expressed widely) and IRE1β (expressed mainly in gastrointestinal tract and lung), IRE1 is a conserved ER type I transmembrane protein with dual activity of protein kinase and ribonucleic acid endonuclease (Karagöz GE et al., 2017). When ERS occurs, IRE1 dissociates from GRP78 and undergoes autophosphorylation, and its ribonucleic acid endonuclease activity is activated to specifically convert X-box binding protein-1 (XBP1) mRNA into active XBP1 splicing (XBP1s). The latter one is translated by ribosomes to form the transcription factor XBP1-s with strong transcriptional activity, which is able to regulate the expression of a variety of protein folding-related genes such as foldase, oxidoreductase, and glycosylase at the level of protein transcription, and promotes endoplasmic reticulum-associated degradation kinase (ERAD) to mitigate ERS (Bartoszewska S et al., 2019). If ERS is not alleviated, IRE1 can activate c-Jun N-terminal (JNK) via tumor necrosis factor receptor associated factors 2 (TRAF2), in the presence of apoptotic signaling kinase (ASK1) signaling pathway to induce cellular autophagy (Li Y et al., 2019). In addition, XBP1, as a target of other proteins during ERS, can alleviate ERS by regulated IRE1α-dependent decay (RIDD) which causes selective degradation of ERS-related proteins (Adams CJ et al., 2019).

### 2.3 ATF6 signaling pathway

ATF6, consisting of the two isoforms ATF6α and ATF6β and other membrane proteins related to ATF6, is an ERS-regulated transmembrane transcription factor whose N-terminal structural domain is in the cytoplasm while the C-terminal is in the ER lumen, and acts to activate the transcription of ER molecular chaperones in order to detect the ERS. Upon ERS onset, GRP78 is dissociated from the N-terminal end of ATF6, allowing ATF6 to be packaged into COPII pericycle vesicles and transported from the ER lumen to the Golgi complex via its Golgi localization sequence. It is subsequently spliced and proteolytically hydrolyzed by Golgi site 1 protease (S1P) and site 2 protease (S2P), converted to the active form p50 ATF6 and translocated to the nucleus where it binds to the ERS response element (ERSE), inducing transcriptional regulation and activating downstream target genes such as GRP78, GRP94 and CHOP (Walter F et al., 2018). In addition, ATF6α and IRE1α can regulate the quantity and quality of XBP1 to fully activate the UPR, relieve ERS and restore ER homeostasis, respectively (Lee K et al., 2002).

**FIGURE 1:**
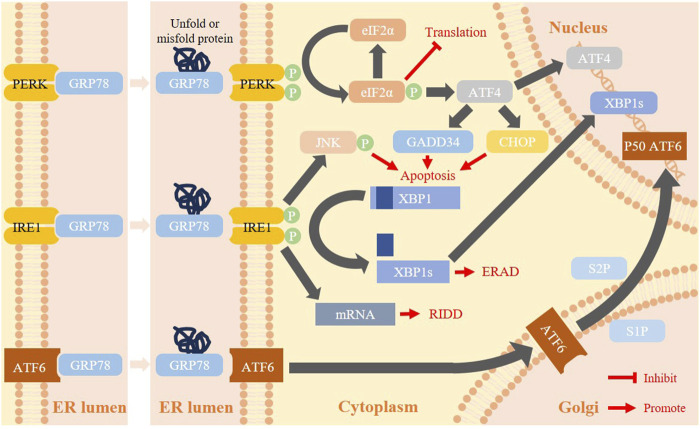
Mechanism of ERS-UPR. When unfolded/misfolded proteins increase due to various causes such as hypoxia, vascular insufficiency, and nutrient deprivation, GRP78 detaches from PERK,IRE1, and ATF4 and binds to them, leading the 3 UPR pathways to shift from an inactivated state (left) to an activated state (right) in order to restore ER homeostasis or to induce autophagy and apoptosis.

When ERS is so severe that the UPR cannot compensate for its damage, cells will initiate cellular autophagy and/or initiate apoptosis mediated by 3 pathways: (1) the CHOP pathway mediated by PREK, IRE1 and ATF6, (2) the IRE1-TRAF2-JNK pathway, and (3) the caspase-12 pathway, thereby removing the abnormal cells. This lethal ERS has been shown to be one of the viable anti-tumor targets (Wu H et al., 2020; Song SE et al., 2023) (Figure 1).

## 3 Dual effect of ERS in digestive system tumors

### 3.1 ERS in oral tumors

Many studies have shown that ERS exerts a predominantly anticancer effect by inhibiting the growth and drug-resistant behavior of tumors from the oral cavity. For example, when human tongue squamous cell carcinoma Tca8113 and CAL-27 cells were in ERS state, expression of tribbles related protein 3 (TRB 3) mRNA and its protein, an important signaling molecule in ERS signaling pathway, was significantly elevated, which could possibly induce apoptosis by inhibiting AKT phosphorylation (Zhang J et al., 2012). Wang J et al. (2019) confirmed that phosphorylation of nuclear factor kappa-B (NF-κB), a crucial transcription factor, mediated by PERK-eIF2α was able to inhibit ERS and thus proliferation and radio resistance of oropharyngeal cancer Fadu and Detroit cells. Lyu X et al. (2019) also reported that IRE1 regulates radio resistance in HPV-negative oropharyngeal carcinoma through activation of IL-6, and enhances X-ray-induced DNA double-strand breaks and apoptosis. Upregulation of GRP78 expression and activation of the PERK-eIF2α-ATF4 pathway via ERS is one of the mechanisms by which an antitumor drug oxaliplatin is able to induce apoptosis of oral squamous carcinoma HSC-3 cells to occur *in vitro* (Li HR et al., 2023).

### 3.2 ERS in EC

EC is divided into two subtypes as esophageal squamous cell carcinoma (ESSC) and esophageal adenocarcinoma (EAC), with the former occupying more than 90% of clinical cases. The important and dual regulatory role played by ERS on early adaption, proliferation and drug resistant survival of EC has been demonstrated.

#### 3.2.1 ERS promotes EC development

Several studies have confirmed the activating and facilitating role of ERS and its downstream responses in EC: Lu H et al. (2022) found that aurora kinase A (AURKA), a mitotic kinases mediating several protumor genic functions, promotes UPR through IRE1 phosphorylation *in vitro* and *in vivo*, resulting in adaptive survival of EAC cells FLO-1, LO33 and SK-GT4. Chen SM et al. (2023) found that *Fusobacterium* nucleatum (Fn)-induced ERS-related proteins GRP78 and XBP1 high expression could promote the malignant evolution of KYSE150 and KYSE140 in ESSC cells. Similarly to it, inhibition of ERAD pathway of ERS was able to inhibit the proliferation of KYSE70 and KYSE140 in EC cells (Luo H et al., 2023). The classical phosphatidylinositol 3-kinase (PI3K)/protein kinase B (Akt) pathway participates in ESSC development by regulating autophagy in tumor cells (Shi H et al., 2023). Research has shown that the phosphatidylinositol 3-kinase (PI3K) inhibitor BEZ235 was able to reverse tunicamycin-induced upregulation of GRP78, PREK, eIFα, CHOP, Bax, and cleaved Caspase-3 in human EC cells EC9706 and EC109, thereby increasing the sensitivity of EC9706 to chemotherapeutic drugs oxaliplatin, inversely demonstrating the role of ERS in increasing drug resistance and reducing tumor cells killed by anticancer drugs in EC cells (Zhou F et al., 2017).

#### 3.2.2 ERS inhibits EC development

However, more research supports the effect of ERS against EC: Huang ZL et al. (2017) found that for ESSC cells EC109, overexpression of maternally expressed gene 3 (MEG28) increased the content of GRP78, IRE1, PERK, ATF6, CHOP and cleaved Caspase-3, and inhibited the proliferation of EC cells *in vitro* by activating ERS and induced apoptosis. Shen ED et al. (2019) reported that tunicamycin-induced ERS activation in human EC cells TE-1 would lead to autophagy and apoptosis, and enhanced the sensitivity of EC cells to anti-tumor drug oxaliplatin, which may be closely related to the regulation of the AKT/mTOR-related pathway. Proteasome 26S subunit non-ATPase 4 (PSMD4) is a proteasome ubiquitin receptor closely related to ERS. It was found that PSMD4 was able to inhibit ERS-mediated apoptosis in human EC cells ECA109 and silencing PSMD4 upregulates GRP78, ATF4 and p-PERK thereby enhancing ERS and exerting anti-tumor effects (Ma AG et al., 2019). DEAD-Box Helicase 5 (DDX5) plays a vital role in RNA metabolism. Ma L et al. (2020) demonstrated that by inhibiting DDX5, downregulation of ERS-related proteins such as BIP, p-eIF2α, p-PERK and P62 promotes cellular autophagy of EC cells TE-1, EC-109, EC-9706, and KYSE-150. Liu Q et al. (2021) revealed that the Bruton tyrosine kinase (BTK) inhibitor ACP-5862 induces cell cycle arrest in EC cells EC-109 and KYSE270 at G2/M phase and cellular apoptosis by upregulating the expressing of ERS-related proteins BIP, ATF4, XBP1, ATF6, CHOP and GADD34. In addition animal experiment showed that the apoptosis could be blocked by ERS inhibiter 4-PBA. Wang C et al. (2020) reported that estradiol activated lethality and apoptosis in EC cells EC-109 by upregulating the expression of GRP78, ATF6, IRE1α and PERK, activating lethal ERS to inhibit EC cells proliferation. These evidence demonstrate the capacity to specifically kill EC cells and exert anticancer effects by modulating the ERS pathway in theory.

### 3.3 ERS in GC

It’s GC that threats human health with low survival and high recurrence rates along with the high incidence, in which ERS affects tumor progression and regression through multiple pathways (YANG and LU, 2021).

#### 3.3.1 ERS promotes GC development

On the one hand, ERS is an important participatory mechanism in the development of GC: genetic study have shown that carriers of the G allele of the autophagy Related 16 Like 1 (ATG16L1) rs2241880 gene have more severe ERS than carriers of the A allele after infection with *Helicobacter pylori* naturally, which exacerbates gastric mucosal lesions of the former (Mommersteeg et al., 2022). Early experiment found that ERS activated the PI3K/AKT pathway and induced epithelial mesenchymal transition in BGC-823 and SGC-7901 cells thereby promoting the migration and invasion of GC cells (Guo, 2017). Another study showed that Sec62 protein also promotes GC cell metastasis by upregulating ERS-associated PERK/ATF4 expression in GC cells and that this effect is attenuated after Sec62 knockdown (Su S et al., 2022). Xing WY et al. (2017) observed that ERS activated the P38 pathway, which induced the development of GC cells BGC-823 and SGC-7901 with doxorubicin resistance. Takahashi S et al. (2023) reported that overexpression of coiled-coil domain containing 85A (CCDC85A) as a target of miR-224-p3 in GC increased the resistance of GC cells to ERS and attenuated the therapeutic effect of cisplatin. J Guo (2018) reported that ERS reduced the sensitivity of GC human epithermal growth factor receptor 2 (HER2)-positive NCI-N87 and MKN-45 cells to the anticancer-targeting agent trastuzumab and attenuated proliferation inhibition, which could be reversed by the ERS inhibitor 4-phenyl butyric acid (4-PBA).

#### 3.3.2 ERS inhibits GC development

However, on the other hand, ERS is also an integral component of the mechanism of GC inhibition: VacA, the causative factor of highly parthenogenic *Helicobacter pylori*, inhibits AGS cell proliferation and mediates autophagy in GC through ERS pathway (Zhu P et al., 2017). BIP, CHOP, and Caspase-3 expression in clindamycin-induced cell SGC-7901 was upregulated, indicating that ERS induced apoptosis in GC cells, and increasing ERS inhibitor 4-PBA could reverse the above effects (Zhang Q and Xu L, 2018). Kong Q et al. (2020) found that microRNA-637 could increase GRP78 and CHOP expression and induced apoptosis in GC cells AGS. Zhou Y et al. (2021) reported that long non-coding RNA nuclear paraspeckle assembly transcript 1 (NEAT1) significantly upregulated ERS-related proteins (XBP-1s/XBP-1U and GRP78) and apoptosis-related proteins (Bax and cleaved Caspase-3), inhibited GC cell proliferation and invasion, and promoted apoptosis. Both vivo and vitro experiments showed that the transcription factor hand and neural crest derivative expressed 1 (HAND1) upregulated expression of BIP, CHOP, ATF6, PERK, ATF4, XBP1-s and IRE1α and synergized with cisplatin in inducing GC cell lines AGS and MKN-28 apoptosis, and knockdown of CHOP attenuated this effect (Kuang Y et al., 2023). Another study showed that silencing the key Calnexin gene in ERAD was able to induce SGC-7901 apoptosis accompanied by an increase in the expression levels of ERS-associated GRP94, IRE1, ATF6, and CHOP proteins (Zhang, XH et al., 2017). A recent study showed that overexpression of the ER-resident protein 44 (ERp44) was able to inhibit proliferation and promote apoptosis of GC cells, MGC-803 and KATO III, by upregulating the eIF2α/CHOP signaling pathway (Tian Y et al., 2023). In drug-resistant SGC-7901/DDP cells, content of miR-34 and miR-7 was significantly lower than that in general GC cells. Further study revealed that upregulation of ATF6, Bip and miR-34 expression and thus regulation of Foxo3a, recombinant p53 upregulated modulator of apoptosis (PUMA) protein expression levels, or upregulation of miR-7 expression and thus regulation of PUMA protein expression levels could promote apoptosis in GC drug-resistant cells (Bi, 2020). Zhao LN et al. (2023) et al. found that vitamin E succinate was able to dose-dependently cause apoptosis in GC cells MKN28, accompanied by an increase in the expression of ERS-associated protein GRP78, autophagy-associated proteins Beclin-1, LC-3II, and apoptosis-associated proteins Caspase-3, which could be reversed by the ERS inhibitor 4-PBA.This suggested that the artificially regulated ERS could play the antitumor role by triggering the autophagy and apoptosis of GC cells.

### 3.4 ERS in HCC

#### 3.4.1 ERS promotes HCC development

Many studies have proved the protective role of ERS in the early survival of HCC: Qu XS et al. (2017) observed that ERS-associated factor XBP-1 was expressed significantly higher in human HCC tissues than that in normal and para-cancerous tissues, which showed that ERS is involved in the development of HCC. Qu TT et al. (2021) reported that overexpression of MAS-related G-protein Coupled Receptor member D (MrgD) can inhibit ERS and fatty acid metabolism in HepG2 cells, suggesting the beneficial role of ERS to HCC. What’s worse, the HCC cells HepG2 and Hep3B under ERS could achieve immune escape by secreting HSP70-rich exosomes, activating the Toll-like receptor (TLR4) signaling pathway and promoting macrophage transformation to the M2 type (Hua W et al., 2021).

Other studies found that ERS is closely related to the invasiveness and anti-tumor drug resistance of HCC as well: early study found that ERS upregulated MMP-9 expression and exacerbated HCC cell SMMC-7721 and HepG2 metastasis and invasion by inducing calreticulin membrane translocation (Zhai W.L. et al., 2015). Mao RT et al. (2018) reported that ERS elevated the expression of HepG2 chemokines CXCL1, CXCL2, and CXCL3, and decreased the expression of CXCL8 in HCC cells, which may have exacerbated the tumor invasion. Wang H et al. (2023) observed that knockdown of FAM134B, a transcriptional response genes to ERS, was able to upregulate the expression of DERL2, EDEM1, SEL1L and HRD1 in HCC cells Hep3B and Huh7, which ultimately reduces apoptosis and promotes tumor growth through the ERS pathway. Moreover, Yan DK et al. (2021) found that inhibition of ERS can increase the sensitivity of HCC cells HepG2 and HuH7 to anticancer drug bortezomib and promote apoptosis of cancer cells, which inversely proves that ERS was able to increase drug resistance and survivability in HCC cells. In line with this, another study showed that HCC cells Hepal-6 and Huh-7 attenuated the anti-tumor ability of cytotoxic T lymphocytes through the ERS pathway and accelerated HCC development (Wang W et al., 2022). J. Cui (2020) revealed that ERS-related transcription factor zinc finger protein 263 (ZNF263) may be one of the potentially relevant super-enhancers of ERS, which is able to not only promote the proliferation and apoptosis resistance of HCC PLC/PRF/5, Hep3B, LM3, SK-HEP-1, HepG2, and Huh7 cells, but also reduce the sensitivity of HCC to sorafenib. Addition of ERS inhibitor 4-PBA or ZNF263 knockdown also confirmed the above effects in reverse.

#### 3.4.2 ERS inhibits HCC development

In contrast to the promotional effect mentioned above, Lu et al. (2017) found that C/EBPβ-3 only express in normal hepatocytes and that ERS-induced upregulation of C/EBPβ-3 might be involved in the death of Hep3B in HCC cells. Vitro experiments demonstrated that anhydrous ethanol-induced ERS significantly upregulated apoptosis, accompanied by increased expression of IRE1, XBP-1, CHOP and Cleaved Caspase-12 (Qi HY et al., 2019). Another study reported that activation of ERS-related proteins ATF6, CHOP and XBP-1 by the PERK/eIF-2α signaling pathway induced release of Caspase12 apoptotic protein and apoptosis in HCC cells (Lin and Wang, 2020).

### 3.5 ERS in CRC

CRC is the digestive tumor with the highest morbidity (10.0%) and mortality (9.4%) nowadays, accounting for about one-tenth of tumor deaths and for which there is no effective clinical therapies worldwide (Cao W et al., 2021). However, there are still relatively few studies of CRC from the ERS pathway.

#### 3.5.1 ERS promotes CRC development

Saini KK et al. (2023) found that RSL3, an inducer of ferroptosis, promotes the expression and phosphorylation of PERK, ATF6 and IRE1α in CRC cells HT29, SW620, DLD1 and HCT116. Additional study revealed that PERK knockdown promotes ferroptosis and inhibits tumor growth *in vitro* and *in vivo*, suggesting that ERS-related protein expression can regulate CRC development. What’s more, Peng H et al. (2022) observed that ERS would promote the proliferation and invasion of CRC cells HCT116, SW480, HT29 and LoVo through the autophagy pathway mediated by ATG5 protein as well. These imply that ERS can accelerate tumor progression by regulating CRC cell programmed death.

#### 3.5.2 ERS inhibits CRC development

Interestingly, early study has observed the decreased expression of ERS-related proteins PERK and ATF6 in CRC cells from clinical patients, suggesting a more complicated relationship between ERS and CRC (Gao L et al., 2017). Rahmani-Kukia N et al. (2023) found that knockdown of endoplasmic reticulum-metallopeptidase 1 (ERMP1) promotes GRP78 translocation in CRC cells HCT116 and SW48, which facilitates G1-phase cycle block and apoptosis. Moreover, the antitumor drug JS-K upregulated p-eIF2α and CHOP proteins and downregulated ATF-4 and Bip proteins in human CRC cells HCT-116, which inhibited the proliferation of cancer cells and induced apoptosis via the ERS pathway (Li YD et al., 2022). Resemble to this, Sun S et al. (2023) observed that ERS activates TAp73α via the PERK-ATF4 pathway, inhibits CRC growth and induces apoptosis in CRC cells HCT116.

### 3.6 ERS in other digestive system tumors

Study have demonstrated that glucose deprivation upregulates GRP78 expression in pancreatic cancer (PDA) cells, implying activated ERS in PDA (Zhao T et al., 2023). Another study reported that squalene epoxidase mitigates ERS by downregulating GRP78 expression and ultimately promotes the proliferation of PDA cells AsPC-1 and BxPC-3 *in vitro* and *in vivo*. Squalene epoxidase inhibitors terbinafine and NB-598 were able to reverse the above effects and to perform anti-tumor effects (Xu R et al., 2023).

Above all, ERS in digestive system tumors can either enhance their migration and drug resistance contributing to the tumor progression or exert antitumor effects by inducing tumor cell death, and the effect varies significantly in different types and stages of tumors. Since ERS hardly occurs in normal cells, selecting appropriate targets to adjust ERS and its downstream pathways according to tumor progression can specifically block or even reverse the development of digestive system tumors compared with contemporary treatment, which may lead to the new breakthrough clinically.

## 4 Chinese botanical drug extracts against digestive system tumors through ERS

Based on thousands of years of application experience, Chinese botanical drug have a great advantage in terms of effectiveness and their extracts have significant therapeutic effects as natural metabolites against digestive system tumors as well. Many studies have demonstrated that Chinese botanical drug extracts exert their antitumor effects by interfering with ERS, especially the 8 terpenoids, 7 flavonoids, 5 quinones, 4 lignans, 4 phenols, 3 alkaloids, 2 coumarins and others mentioned below (Table 1).

**TABLE 1:** Chinese botanical drug extracts against digestive system tumors through ERS.

Tumor	Extract	Origination	Structure	Model	Biological effects	Results	References
Oral tumor	Gambogenic acid	*Garcinia hanburyi* Hook.f	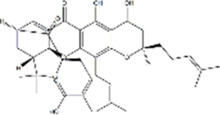	CNE-2Z cells	CHOP↑ ATF4↑	Inhibits tumor growth	Xu T and Li QL (2017)
					GRP78↓	Induces apoptosis	
EC	Icariin	*Epimedium brevicornu* Maxim	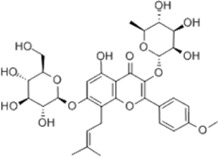	EC109/TE1 ESCC cells	p-PERK↑ GRP78↑ ATF4↑ p-eIF2α↑ CHOP↑ PUMA↑	Blocks cell migration	Fan C et al. (2016)
						Induces apoptosis	
	Corilagin	*Punica granatum* L	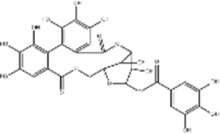	ECA-109/KYSE150 cells	Cleaved Caspase-12↑ Cleaved Caspase-7↑	Blocks cell migration	Wu C et al. (2021)
					GRP78↓	Blocks cell cycle at G0/G1	
						Induces apoptosis	
	TanshinoneⅡA	*Salvia miltiorrhiza* Bunge	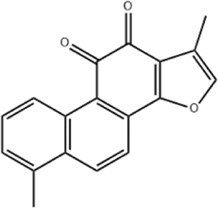	ECA109 cells	Caspase-4 Caspase-9↑CHOP↑	Induces apoptosis	Zhu YQ et al. (2016), Zhang Y et al. (2017)
					BIP↓		
	Acetylshikonin	*Arnebia euchroma* (Royle) Johnst./*Lithospermum erythrorhizon* Sieb. et Zucc./*Arnebia guttata* Bunge	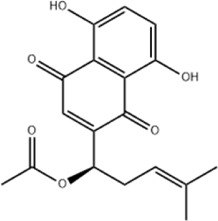	KYSE450/TE10/KYSE180/KYSE510/EC109/EC9706 cells, mice	BIP↑ ATF3↑ ATF4↑ p-eIF2α↑ XBP-1↑	Inhibits tumor growth	Yuan YJ et al. (2023)
						Blocks cell cycle at G1/S	
						Induces apoptosis	
	α-asarone	*Acorus tatarinowii* Schott	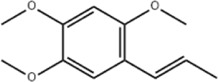	Eca-109 cells	GRP78↑ CHOP↑	Inhibits tumor growth	Shi JJ et al. (2017), Li H et al. (2022)
					Caspase-4↓ BIP↓	Induces apoptosis	
GC	Wogonoside	*Scutellaria baicalensis* Georgi	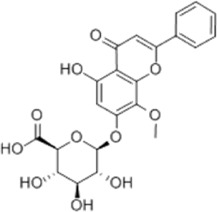	AGS/MKN-45 cells	CHOP↑ GRP78↑ GRP94↑ IRE1α↑ TRAF1↑ p-ASK↑ p-JNK↑	Reduces cells viability	Gu Q et al. (2021)
						Blocks cell cycle	
						Induces apoptosis	
	Kaempferol	*Kaempferia galanga* L	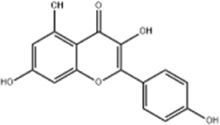	AGS/SNU-216/NCI-N87/SNU-638/NUGC-3/MKN-74 cells	IRE1↑ p-IRE1↑ p-JNK↑ CHOP↑ Cleaved Caspase-12↑	Inhibits cells viability	Kim TW et al. (2018)
						Induces apoptosis	
	Baicalein	*Scutellaria baicalensis* Georgi/*Oroxylum indicum* (L.) Vent	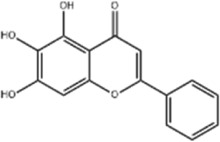	HGC-27/AGS cells, mice	GRP78↑ CHOP↑	Inhibits cells proliferation	Shen J et al. (2023)
						Blocks cell cycle at G0/G1	
	Isoquercitrin	*Follum* Mori	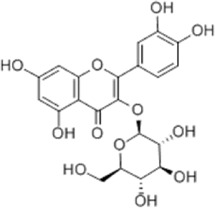	HGC-27/AGS/MKN-45/SNU-1 cells	BAX↑ Cleaved Caspase-3↑ Caspase-12↑	Inhibits cell proliferation	Liu J et al. (2023)
	Nobiletin	*Citrus reticulata* Blanco	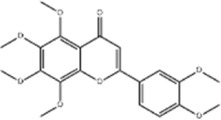	HGC-27/MKN-28 cells	--------	Inhibits cell proliferation	Chen M et al. (2023)
	Salidroside	*Rhodiola crenulata* (Hook. f. and Thomson) H. Ohba	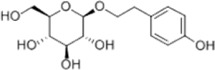	SGC-7901 cells, mice	CHOP↑ Cleaved Caspase-12↑	Inhibits cells growth and migration	Yan W et al. (2019)
					GADD34↓ BIP↓	Induces apoptosis	
	Saikosaponin A	*Bupleurum scorzonerifolium* Willd./*Bupleurum chinense* DC.	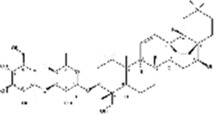	AGS/SNU-638/SNU-216/MKN-74/MKN-7/NCI-N87 cells, mice	BCL-2↓	Inhibits cell viability	Kim (2023)
						Induces apoptosis	
						Reduces radiation resistance	
	Cinnamaldehyde	*Cinnamomum cassia* Presl	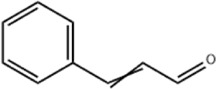	SNU-638/SNU-216/AGS/NCI-N87/MKN-45/MKN-74 cells	GRP78↑ p-PERK↑ p-eIF2α↑ CHOP↑	Inhibit cells proliferation	Kim (2022)
						Induces apoptosis	
	Chicoric acid	*Echinacea purpurea* (L.) Moench	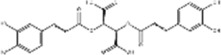	SGC-7901/MGC-803 cells, mice	CHOP↑ BIP↑ Parkin↑ p-PERK↑ PERK↑ p-IREα↑ IREα↑ p-eIF2α↑ ATF4↑ ATF6↑	Induces apoptosis	Sun X et al. (2019)
	Oxymatrine	*Sophora flavescens* Aiton	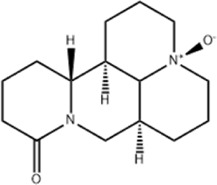	BGC-823 cells	GRP78↑ BIP↑ Caspase-12↑	Inhibits cell proliferation	Fang et al. (2019)
						Induces apoptosis	
	Piperlongumine	*Piper nigrum* L	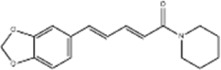	SGC-7901/BGC-823 cells, mice	p-eIF2α↑ ATF4↑ CHOP↑	Inhibits cell growth	Zou P et al. (2016)
						Blocks cell cycle	
						Induces apoptosis	
	Berbamine	*Phellodendron chinense* C.K.Schneid./*Phellodendron amurense* Rupr	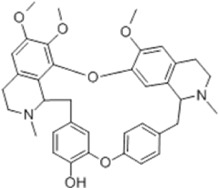	AGS cells	BAX↑ CHOP↑ GRP78↑	Induces apoptosis	Xu H et al. (2022)
					Cleaved Caspase-12↑		
					BCL-2↓		
	Allicin	*Allium sativum* L	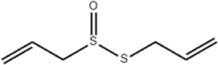	SGC-7901 cells	GRP78↑ PERK↑ p-PERK↑ eIF2α↑ ATF-4↑ CHOP↑	Inhibits cell proliferation	Wu SF and Li ZJ (2019)
						Induces apoptosis	
	Myristicin	*Myristica fragrans* Houtt	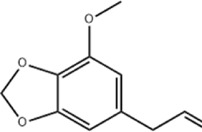	AGS/MKN-45 cells	GRP78↑ ATF6↑	Inhibits tumor growth	Song J et al. (2023)
	Esculetin	*Fraxinus rhynchophylla* Hance	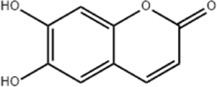	SGC-7901 cells	GRP78↑ p-PERK↑ p-eIF2α↑ ATF-4↑ CHOP↑	Induces apoptosis	Yan TH (2020)
	Schizandrin A	*Schisandra chinensis* (Turcz.) Baill	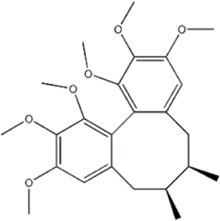	GES-1/AGS cells	p-PERK↑ p-eIF2α↑ CHOP↑	Inhibits tumor viability	Pu H et al. (2021)
						Blocks cell cycle	
						Induces apoptosis	
	Schizandrin B	*Schisandra chinensis* (Turcz.) Baill	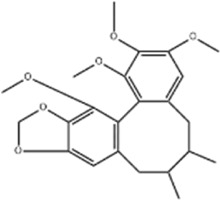	BGC-823 cells	Bax↑ Caspase-3↑ CHOP↑ p-PERK↑ p-eIF2α↑ ATF4↑	Induces apoptosis	Ge YN et al. (2022)
					p-PI3K↓ p-Akt↓		
	Resveratrol	*Reynoutria japonica* Houtt	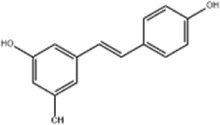	AGS cells	GRP78↑ PERK↑ p-eIF2α↑ CHOP↑ Cleaved Caspase-12↑	Blocks cell cycle at G2/M	Ren M et al. (2020)
						Induces apoptosis	
	RTR-1	*Rhodomyrtus tomentosa* (Aiton) Hassk	--------	BGC-823/SGC-7901 cells	IRE1α↑ CHOP↑ PERK↑ BIP↑	Inhibits cell growth	Zhang X et al. (2020)
						Blocks cell cycle	
						Induces apoptosis	
	Ethanolic extract of Cordyceps cicadae	Cordyceps cicadae	--------	SGC-7901 cells	Bax↑ AIF↑ Caspase-8↑ Caspase-6↑ Caspase-3↑	Blocks cell cycle at S	Xie H et al. (2019)
						Induces apoptosis	
HCC	Youchasaponin	*Camellia oleifera* Abel	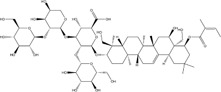	HepG2 cells	IRE1↑ PERk↑ eIF2α↑ eIF2α↑	Induces apoptosis	Ma LY et al. (2011)
	Ginsenoside CK	*Panax ginseng* C. A. Mey./*Panax notoginseng* (Burkill) F. H. Chen ex C. H. Chow	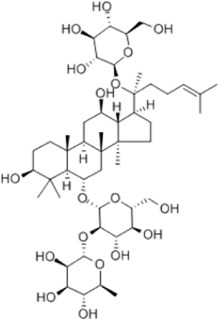	SMMC-7721 cells, mice	GRP78↑ p-IF2α↑ p-JNK↑ Caspase4↑ CHOP↑	Induces apoptosis	Lv L (2019)
						Inhibit tumor growth	
				HepG2 cells	Cleaved Caspase3↑ Cleaved PARP↑ GRP78↑ CHOP↑ Cleaved Caspase 4↑ p-PERK↑ p-IRE1↑ p-JNK↑ p-eIF2α↑	Induces apoptosis	Chen JX et al. (2020)
					p-STAT3↓		
	Glycyrrhetinic acid	*Glycyrrhiza uralensis* Fisch. ex DC./*Glycyrrhiza inflata* Batalin/*Glycyrrhiza glabra* L	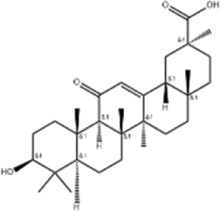	SMMC-7721/HepG2 cells, mice	CHOP↑ LC3BII↑	Blocks cell cycle at G0/G1	Chen J (2019)
						Induces apoptosis and autophagy	
	Celastrol	*Tripterygium wilfi* Hook F	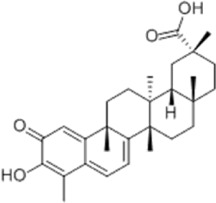	HepG2/Bel7402 cells, mice	p-elF2↑ p-IRE1↑ BIP↑ ATF4↑ CHOP↑ XBP1s↑	Inhibits cell viability	Ren B (2018)
						Blocks cell cycle at G2/M	
						Induces apoptosis	
	Cryptotanshinone	*Salvia miltiorrhiza* Bge	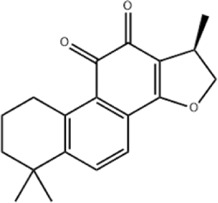	HepG2 cells	Cleaved Caspase-3↑	Induces apoptosis	Wang ZL et al. (2022)
					BIP↓ p-PERK↓		
	Quercetin	*Styphnolobium japonicum* (L.) Schott	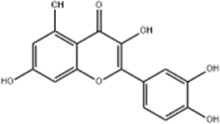	HepG2 cells	GRP78 Snail↓	Inhibits tumor growth and proferliferation	Li SS et al. (2017)
	Osthole	*Cnidium monnieri* (L.)Cuss	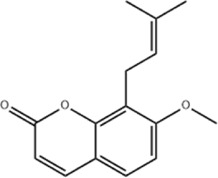	SMMC7721 cells	GRP78↑ CHOP↑ ATF-4↑ GADD34↑ XBP-1s↑	Inhibits cell proliferation	Pan et al. (2019)
					BAX↓	Blocks cell cycle at G1	
						Induces apoptosis	
	Curcumin	*Curcuma longa* L	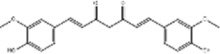	SMMC-7721 cells	GRP78↑ Caspase-12↑ CHOP↑ p-elF2α↑ p-JNK↑ Caspase-4↑	Inhibits cell proliferation	Li YY and Lu MJ (2020)
						Induces apoptosis	Zhang et al. (2021)
	Arctigenin	*Arctium lappa* L	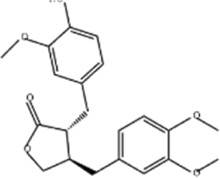	SMMC-7721 cells	GRP78↑ CHOP↑	Induces apoptosis	Chang L et al. (2020)
					Caspase-12↑		
	Tannic acid	*Rhus potaninii* Maxim	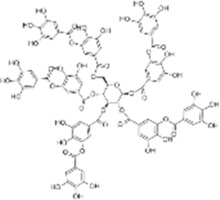	HepG2 cells	GRP78↑ GRP94↑	Inhibits cell proliferation	Geng NN et al. (2018)
						Induces apoptosis	
	Furanocoumarin	*Hansenia weberbaueriana* (Fedde ex H. Wolff) Pimenov and Kljuykov	--------	HepJ5/Mahlavu cells, mice	PERK↑ CHOP↑	Inhibits cell viability	Huang TY et al. (2023)
						Induces apoptosis	
	Licochalcone B	*Glycyrrhiza inflata* Batalin	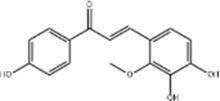	HepG2/Huh7 cells	--------	Inhibits cell viability	Zhang YY et al. (2022)
	Saponin fraction from Gleditsia sinensis	*Gleditsia sinensis* Lam	--------	H22 cells	GRP78↑ CHOP↑	Inhibits cell proliferation	Yin C et al. (2019)
						Induces apoptosis	
CRC	Cucurbitacin	*Cucumis melo* L	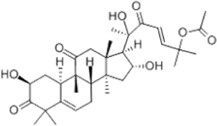	HT-29 cells	p-PERK↑ p-eIF2α↑ ATF4↑ IRE1α↑ XBP1↑ CHOP↑ GRP78↑	Inhibits cell proliferation	Huang X et al. (2023)
						Induces apoptosis	
	Oridonin	*Rabdosia rubescens* (Hemsl.)Hara	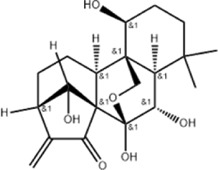	NCM460/HCT116/LoVo/SW480/RKO/HeLa/PC-3/A594 cells, mice	ATF4↑ CHOP↑	Induces apoptosis	Zhou F et al. (2023)
	Emodin	*Rheum officinale* Baill	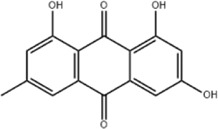	SW620 cells	GRP78↑ CHOP↑	Inhibits cell proliferation	Liu BR et al. (2016)
					Cleaved Caspase-12↑	Induces apoptosis	
	Konjac glucomannan	*Amorphophallus konjac* K.Koch/*Amorphophallus variabilis* Blume	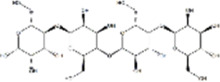	HCT-8 cells	Cleaved Caspase-3↑BAX↑ PERK↑	Inhibits tumor growth	Lu MJ and Chu WJ (2021)
					Cleaved caspase-9↑ ATF4↑ CHOP↑	Blocks cell cycle	
					BCL-2↓	Induces apoptosis reduces radiation resistance	
	Bufalin	*Bufo gargarizans* Cantor/*Bufo melanostictus* Schneider	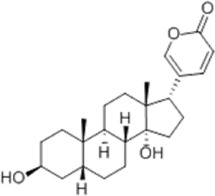	HCT116 cells	BAX↑ GRP78↑ p-PERK↑ p-eIF2α↑ CHOP↑	Induces apoptosis	Shang J et al. (2023)
					BCL-2↓ eIF2α↓	Reduces radiation resistance	
	Dehydrodiisoeugenol	*Myristica fragrans* Houtt	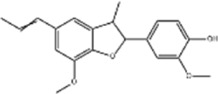	HCT116/SW620 cells, mice	BIP↑ Ero2-Lα↑ PERK↑ eIF2α↑ CHOP↑ p-eIF1α↑ IRE1α↑ XBP-48s↑	Inhibits cell proliferation and tumor growth	Li YD et al. (2022)
						Blocks cell cycle at G1/S	
						Induces autophagy	
	Gambogenic acid	*Garcinia hanburyi* Hook.f	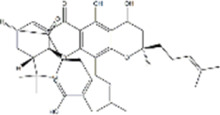	HCT116 cells	------	Inhibits cell proliferation	TT Dai et al. (2014)
						Induces apoptosis	
	Osthole	*Cnidium monnieri* (L.)Cuss	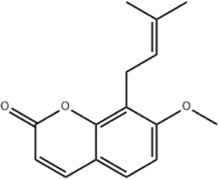	HT-29 cells	p-PERK/PERK↑ p-elF2α/elF2α↑	Inhibits cell proliferation	Zhou XH et al. (2021)
					GRP78↑ CHOP↑	Induces apoptosis	
	Curcumin	*Curcuma longa* L	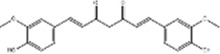	LoVo/HCT-29 cells	BIP↑ PDI↑ CHOP↑	Blocks cell cycle at S	Huang YF et al. (2017) Zhu C et al. (2022)
						Induces apoptosis	
Others	Usnic acid	*Usnea diffracta* Vain	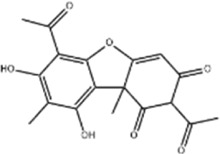	MIA PaCa-2/PANC-1 cells	BIP↑ IRE1α↑ GADD153↑	Inhibits tumor growth	Gimła M et al. (2023)
						Blocks cell cycle	
						Induces apoptosis reduces radiation resistance	
	Oridonin	*Rabdosia rubescens* (Hemsl.)Hara	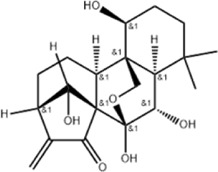	SW 1990/Panc-1 cells, mice	Cleaved Caspase-12↑ GRP78↑ CHOP↑	Inhibits cell proliferation	Liu DL et al. (2022)
						Induces apoptosis	

### 4.1 Esophageal cancer

Icariin, a flavonoid glycoside from botanical drugs such as *Epimedium brevicornu* Maxim, has been used in the treatment of a variety of tumors such as prostate cancer (Chen C et al., 2023). Fan C et al. (2016) reported that icariin (20, 40, 80 μM for 12, 24, 36 h) dose-dependently upregulated the expression of p-PERK, GRP78, ATF4, p-eIF2α, CHOP and pro-apoptotic protein PUMA and downregulated the expression of anti-apoptotic protein Bcl2 in human EC cells EC109 and TE1 ESCC, ultimately interfering tumor cell adhesion and migration, which were attenuated after inhibition of ERS by eIF2α siRNA.

Corilagin is a quinone extracted from many botanical drug such as *Punica granatum* L. with effects such as anticoagulant and antitumor (Wang X et al., 2023). Wu C et al. (2021) found that corilagin (10, 20, 40 μM for 24 h) was able to time- and dose-dependently upregulate cleaved Caspase-7 and cleaved Caspase-12 expression, to downregulate GRP78 expression, to inhibit ESC cell ECA-109, KYSE150 and HEEPIC migration, blocking the cell cycle at G0/G1 phase and promoting apoptosis *in vitro* and *ex vivo*.

Tanshinone IIA is a quinone metabolite extracted from the botanical drug *Salvia miltiorrhiza* Bge, which has anti-inflammatory and cardioprotective effects (Xu Q et al., 2023). Zhu YQ et al. (2016) reported that tanshinone IIA (2, 4, 8 μg/mL for 12, 24, 36 h) was able to induce apoptosis by upregulating Caspase-4 and CHOP levels in human EC cells ECA109 in a time- and dose-dependent manner. Further study confirmed that tanshinone IIA (25, 40, 60 μg/mL for 24h, 48h, 72 h) induced death of EC109 by dose-dependent upregulation of Caspase-9, downregulation of BIP and then activation of CHOP (Zhang Y et al., 2017).

Acetylshikonin is a quinone with strong anticancer effect isolated from the botanical drug *Lithospermum erythrorhizon* Sieb. et Zucc. (Cha HS et al., 2023). Yuan YJ et al. (2023) reported that acetylshikonin (10 mM for 24 h) inhibited the proliferation of ESCC cells *in vitro* at KYSE450, TE10, KYSE180, KYSE510, EC109, EC9706 and blocked cell cycle in G1/S phase. Additional experiments *in vitro* and *in vivo* revealed that increased apoptosis in KYSE180 and KYSE450 cells was accompanied with upregulation of BIP, ATF3, ATF4, p-eIF2α, and XBP-1 and was attenuated by CHOP knockdown and the PERK inhibitor GSK2606414, which demonstrated that acetylshikonin inhibits ESCC through activation of the PERK/eIF2α/CHOP axis.

α-asarone is a phenylpropanoid extracted from the botanical drug *Acorus tatarinowii* Schott and serves effectively in several systems’ diseases with antilipidemic, antioxidant and anti-inflammatory effects (Shi et al., 2021; Fan et al., 2022). Early study has found that α-asarone (25, 50, 100 μg/mL for 12h, 24h, 36h, 48 h) exerts an inhibitory effect on the proliferation of human EC cells ECA-109 by decreasing the expression of Caspase-4 mRNA and protein, and increasing the expression of GRP78 mRNA and protein (Shi JJ et al., 2017). A recent study revealed that α-asarone (25, 50, 100 mg/L for 48 h) was sufficient to dose-dependently upregulate the expression of GRP78 and CHOP proteins, downregulate the expression of BIP, significantly promoted apoptosis and inhibited proliferation of Eca-109 cells (Li H et al., 2022).

### 4.2 Gastric cancer

Wogonoside, a major metabolite of the botanical drug *Scutellaria baicalensis* Georgi, is a flavonoid with antitumor and other effects (Luo M et al., 2018). Gu Q et al. (2021) found that wogonoside (10, 25, 50, 75 μM for 0, 6, 12, 24, 48, 72, 96 h) significantly upregulated expression of CHOP, GRP78, GRP94, IRE1α, TRAF1, p-ASK and p-JNK in AGS and MKN-45 cells, reduced cell viability and induced apoptosis in GC cells, and its biological effects could be blocked by IRE1α knockdown.

Kaempferol is a flavonoid widely distributed in botanical drug *Kaempferia galanga* Lt and various types of fruits and vegetables, having a strong anticancer ability (Guo HQ et al., 2021). It has been reported that kaempferol (50 μM for 8, 16, 24 h) was able to inhibit AGS, SNU-216, NCI-N87, SNU-638, NUGC-3 and MKN-74 cells’ viability. Further study revealed that kaempferol promoted apoptosis in GC cells via the IRE1-JNK-CHOP signal pathway by upregulating the expression of IRE1, p-IRE1, p-JNK, CHOP and cleaved Caspase-12 in AGS and SUN-638. This effect was blocked upon inhibition of autophagy or IRE1 (Kim TW et al., 2018).

Baicalein, a flavonoid extracted from the root of the botanical drug *Scutellaria baicalensis* Georgi or *Oroxylum indicum* (L.) Vent., protects cells and fights against a variety of tumors (Gan YC et al., 2023). Shen J et al. (2023) found that baicalein (30, 60, 120 μM for 48 h) dose-dependently upregulated the expression of GRP78 and CHOP and blocked the PI3K/AKT pathway to trigger the ERS, blocking the cell cycle at G0/G1 phase and inhibited the proliferation of GC HGC-27 and AGS cells. The above effects were reversed with the addition of ERS inhibitor 4-PBA. *In vivo* experiments also confirmed that GC progression was significantly prevented in AGS subcutaneous xenograft mice treated with baicalein (15, 50 mg/kg/d for 4 weeks).

Isoquercitrin is a flavonoid widely found in many botanical drugs such as *Follum* Mori, which has pro-healing and anti-tumor effects (Wang YB et al., 2023). Liu J et al. (2023) reported that isoquercitrin showed to be effective in the treatment of GC cell lines such as HGC-27, AGS, MKN-45 and SNU-1 by upregulating BAX, cleaved Caspase-3 and Caspase-12, and downregulating BCL-2.

Nobiletin, a flavonoid from the botanical drug *Pericarpium Citri* Reticulatae and its variants, has a variety of biological effects such as antithrombotic, anti-inflammatory and antioxidant (Geng YZ et al., 2023; Huang L et al., 2023). Chen M et al. (2023) found that nobiletin inhibited the proliferation of GC cells HGC-27 and MKN-28, which may be mediated through the activation of IRE-1α/GRP78/CHOP axis.

Salidroside is a glucoside extracted from the botanical drug *Rhodiola crenulata* (Hook. f. and Thomson) H. Ohba with proved anticancer effects (Zhang QL et al., 2023). Yan W et al. (2019) reported that salidroside (1,2.5,5,10,25,50,100,250 μM for 48 h) was able to dose-dependently upregulate CHOP and cleaved Caspase-12 levels and significantly downregulate GADD34 and BiP levels in SGC-7901 cells, inhibiting GC cell growth and migration and PI3K/Akt/mTOR thereby promoting cancer cell apoptosis, which can be blocked by autophagy inhibitors. Animal experiments also confirmed that salidroside (50 mg/kg/2d for 1 week) inhibited GC growth in tumor-bearing mice.

Saikosaponin A is a triterpenoid derived from botanical drug *Bupleurum scorzonerifolium* Willd. or *Bupleurum chinense* DC. and has inhibitory effects on HCC, nasopharyngeal carcinoma and lung cancer (Gao W and Wang JY, 2023). Kim et al. (2018) reported that saikosaponin A (1, 2.5, 5, 10, 20 μM for 24 h; 0, 8, 16, 24 h, 10 μM) inhibited the viability of GC cell lines AGS, SNU-638, SNU-216, MKN-74, MKN-7 and NCI-N87. Additional study revealed that saikosaponin A induced apoptosis by upregulating the levels of GRP78, p-PERK, p-eIF2α, ATF4, and CHOP in AGS and MKN-74, and overcome radio resistance in GC cells thus, a process that could be blocked by GRP78 or PERK knockdown.

Cinnamaldehyde is an aldehyde metabolite from botanical drug *Cinnamomum cassia* Presl, which has good antibacterial and anticancer effects (Wu, J.D. et al., 2023; Tang, T, et al., 2023). Kim et al. (2018) revealed that cinnamaldehyde(10, 25, 50, 100 μg/mL for 0, 8, 16, 24 h) inhibited the cellular growth of human GC cells SNU-638, SNU-216, AGS, NCI-N87 MKN-45 and MKN-74. What’s more, study also showed that cinnamaldehyde induced autophagic GC cell death by increasing the expression of GRP78, p-PERK, p-eIF2α and CHOP in GC cells NCI-N78 and MKN-74, a result that could be blocked by PERK or CHOP knockdown.

Chicoric acid, a phenolic compound, is the main active metabolite of botanical drug *Echinacea purpurea* (L.) Moench and has antioxidant and antitumor effects (Gao HY et al., 2022). Sun X et al. (2019) found that chicoric acid dose-dependently and significantly increased the expression of CHOP, BIP, Parkin, p-PERK, PERK, p-IREα, IREα, p-eIF2α, ATF4, and ATF6 in human GC cells SGC-7901 and MGC-803 (5, 10, 20, 40, 80, 100 μM for 24, 48 h) and SGC-7901 constructed xenograft mice (12.5, 25, 50 mg/kg/2d for 27d), induced apoptosis by upregulating level of p70s6k signal thereby activating the AMPK signaling pathway, and had anticancer effects both *in vivo* and *ex vivo*, an effect that was blocked by the ERS blocker 4-PBA.

Oxymatrine from the root of the botanical drug *Sophora flavescens* Aiton has anti-inflammatory and antioxidant effects and is an alkaloid able to ameliorate a variety of diseases of the cardiovascular system and digestive system (Huang X et al., 2023; Xu DR et al., 2023). Fang W et al. (2019) observed that oxymatrine (30, 60, 90 μmol/L for 24, 48, 72 h) upregulated intracellular GRP78/Bip and Caspase-12 gene and protein expression levels, inhibited cancer cell growth, and induced Caspase-12-dependent ERS apoptosis and G2/M phase cell block in GC cells BGC-823 in a time- and concentration-dependent manner. The application of the ERS inhibitor salubrinal was able to inhibit the above effects, proving that oxymatrine functions through the ERS apoptosis.

Berbamine is a flavonoid widely distributed in *Phellodendron chinense* C.K.Schneid. , *Phellodendron amurense* Rupr. and other botanical drugs with anti-inflammatory and anti-tumor effects (Chen Y et al., 2022; Tao L and Yan JD, 2023). Xu H et al. (2022) reported that berbamine(10, 20, 40, 80, 120, 160 μg/mL for 48 h) induced apoptosis in GC cells AGS via ERS pathway by dose-dependently upregulating the expression of Bax, CHOP, GRP78 and cleaved Caspase-12 and downregulating the expression level of Bcl-2.

Allicin is a propyl ether from botanical drug *Allium sativum* L. with good antibacterial, anti-inflammatory, and anticancer effects (Cai YY et al., 2023; Wu JL et al., 2023). Wu SF and Li ZJ (2019) reported that allicin (12, 24, 48 μg/mL for 24, 48, 72 h) inhibited the proliferation of GC cells SGC-7901and promoted their apoptosis by dose-dependently increasing the expression of GRP78, PERK, p-PERK, eIF2a, ATF-4 and CHOP.

Myristicin is an extract from the botanical drug *Myristica fragrans* Houtt, which has anti-inflammatory, antioxidant and other effects (Ismail Abo El-Fadl and Mohamed, 2022). Song J et al. (2023) found that myristicin (7.8125, 15.625, 31.25 μM for 48 h) inhibited the growth of AGS and MKN-45 cells by increasing the expression of GRP78 and ATF6 and suppressing the EGFR/ERK signaling pathway.

Esculetin, a type of coumarin, is the main active metabolite of botanical drug *Fraxinus rhynchophylla* Hance with anti-cellular-damage effect (Cheng T et al., 2021). Yan TH (2020) found that esculetin (0.28, 0.56,1.12 mmol/L for 48 h) dose-dependently upregulated GRP78, p-PERK, p-eIF2α, ATF-4, and CHOP expression in human GC cells SGC-7901, and this effect would be reduced by the ERS inhibitor Tauro ursodeoxycholic acid (TUDCA), implying that esculetin promotes apoptosis of tumor cells through oxidative stress with ERS.

Schizandrin A and schizandrin B are both active lignan metabolites extracted from the botanical drug *Schisandra chinensis* (Turcz.) Baill with antioxidant and antitumor effects (Xiang LB et al., 2023; Zeng J et al., 2023). Pu H et al. (2021) found that schizandrin A (10, 20, 30, 40, 50 μg/mL for 24, 48, 72 h) was able to upregulate the expression of p-PERK, p-eIF2α, and CHOP in GES-1 and AGS cells, inhibiting GC cells’ viability and inducing apoptosis, which could be reversed by the ERS inhibitor 4-PBA. Another study demonstrated that schizandrin B (12.5, 25, 50, 100, 150, 200 μmol/L for 24 h) inhibited tumor cell viability and induced apoptosis through the upregulation of Bax, Caspase-3, CHOP, p-PERK, p-eIF2α, and ATF4 expression, and downregulation of p-PI3K and p-Akt expression to regulate ERS and promote apoptosis in GC cells BGC-823 (Ge YN et al., 2022).

Resveratrol is a polyphenolic metabolite distributed in a variety of botanical drugs such as *Reynoutria japonica* Houtt. with antioxidant, anti-inflammatory and other effects (Liu LJ and Zheng HJ, 2023). Ren M et al. (2020) found that resveratrol (10, 20, 30, 40, 50, 60, 70, 80 μM for 24, 48, 72 h) could synergize with chemotherapeutic drug cisplatin to significantly increase the expression of GRP78, PERK, p-eIF2α, CHOP and cleaved Caspase-12 in GC cells AGS, blocking the cell cycle at G2/M phase and inducing apoptosis.

RTR-1, an active metabolite from the root of *Rhodomyrtus tomentosa* (Aiton) Hassk., is a potent antioxidant (Saising J and Voravuthikunchai SP, 2012). Zhang X et al. (2020) reported that RTR-1 (3.12, 6.25, 12.5, 25, 50, 100 μmol/L for 48 h) was able to increase the expression of IRE1α, CHOP, PERK, and BiP proteins in GC cells BGC-823 and SGC-7901, to block the cell cycle, to inhibit cancer cell growth and to induce apoptosis.

Ethanolic extract of Cordyceps cicadae comes from the complex formed by the zygote and its larvae parasitized by the *fungus Cordyceps sinensis* (BerK.) Sacc on the larvae of Bat Moths, a widely used botanical drug, and its extracts have immune-enhancing, anti-inflammatory, and antioxidant effects (Xing SL et al., 2023). Study showed that its ethanol extract was able to dose-dependently increase Bax, AIF, caspase-8, caspase-6 and caspase-3 activities and decrease Bcl-2 activity in human GC cells SGC-7901. It also upregulated the expression of calpain-1, caspase-12 and caspase-9, blocking the cells at S phase and exerting anti-tumor effects (Xie H et al., 2019).

### 4.3 Hepatocellular carcinoma

Youchasaponin is a triterpenoid saponin extracted from *Camellia oleifera* Abel, which has the efficacy of lowering blood lipids, relieving cough and calming asthma (Zhou GH et al., 2021). Ma LY et al. (2011) found that youchasaponin (10, 20, 30 μg/mL for 24 h) induced apoptosis in human HCC cells HepG2 by upregulating the expression of IRE1, PERK, eIF2α and eEF2 proteins.

Ginsenoside CK is a triterpenoid derivative extracted from the botanical drugs *Panax ginseng* C. A. Mey and *Panax notoginseng* (Burkill) F. H. Chen ex C. H. Chow, which has anti-inflammatory, antioxidant, and other effects (Yang et al., 2022; Liu J et al., 2023). Lv (2019) found that ginsenoside CK (5, 10, 20 mg/kg/d for 15 d) was able to dose-dependently induce apoptosis and inhibit tumor growth in SMMC-7721 cells and xenograft mice by up-regulating the expression of GRP78, p-IF2α, p-JNK, Caspase4 and CHOP. Chen JX et al. (2020) further revealed that ginsenosides CK(20, 40, 60 μg/mL for 24, 48, 72 h) induced apoptosis in HCC cells HepG2 by upregulating the expression of cleaved Caspase3, cleaved PARP, GRP78, CHOP, cleaved Caspase 4, p-PERK, p-IRE1, p-JNK and p-eIF2α, and downregulating the expression of p-STAT3.

Glycyrrhetinic acid is a triterpenoid derived from the root of the botanical drug *Glycyrrhiza uralensis* Fisch. ex DC., *Glycyrrhiza inflata* Batalin or *Glycyrrhiza glabra* L., which has anti-inflammatory and antitussive effects (Li Y et al., 2023). Chen J (2019) found that 18β-glycyrrhetinic acid was able to promote the expression of ATF4, CHOP, IRE1, XBP1s and LC3BII *in vivo* (25, 50 mg/kg/d for 1 month) and *in vitro* (100,150,200 μM for 24 h), to block the cell cycle at G0/G1 through ERS, and to induce apoptosis and autophagy in HCC cells SMMC-7721 and HepG2. The siRNA knockdown experiment on ATF4, CHOP, and IRE1 demonstrated that the above effects were realized by the ATF4/CHOP pathway.

Celastrol is a triterpenoid extracted from the root of the botanical drug *Tripterygium wilf. i* Hook F and is able to fight against a variety of tumors (Xu and Li, 2017; Yu B et al., 2022). Ren B (2018) found that celastrol upregulated the expression levels of p-elF2α and p-IRE1, Bip, ATF4, CHOP, and XBP1s in HCC cells HepG2 and Bel7402, to undergo G2/M-phase cell cycle block, decreased viability, and then induced apoptosis.

Cryptotanshinone is a diterpene derived from the root of the botanical drug *Salvia miltiorrhiza* Bge, which has a variety of biological effects such as anti-inflammatory and anti-tumor (Liu JL et al., 2021; Niu and Zhang, 2023). Wang ZL et al. (2022) reported that cryptotanshinone (10, 15, 30 μmol/L for 48 h) significantly upregulated cleaved caspase-3 expression, decreased BIP and p-PERK expression, inhibit ERS, and promoted apoptosis in HepG2 cells.

Quercetin is a flavonoid from botanical drugs such as *Styphnolobium japonicum* (L.) Schott with anti-inflammatory, antioxidant, and estrogen-like effects (Wang YT et al., 2023). Quercetin (12.5, 25, 50, 100, 200, 400 μmol/L for 48 h) was found to cause a decrease in GRP78, Snail, and Vimentin mRNA and protein expression levels in HCC cells HepG2, inhibiting ERS and its epithelial mesenchymal transition to suppress the proliferation and metastasis of tumor cells (Li SS et al., 2017).

Arctigenin is a lignan-like metabolite distributed in botanical drugs such as *Arctium lappa* L. with antioxidant and antitumor effects (Zhao J et al., 2023). Study have shown that arctigenin (10, 50, 100 μmol/L for 48 h) dose-dependently upregulate the expression of GRP78, CHOP, and caspase-12, and induce apoptosis of HCC cells SMMC-7721 *in vitro* (Chang L et al., 2020).

Tannic acid is a polyphenolic metabolite distributed in a variety of food crops such as *Rhus potaninii* Maxim., with antibacterial, antitumor, and other bioactivities (Xie Y et al., 2023). Geng NN et al. (2018) reported that tannic acid (90, 180, 270, 360, 450, 540 μmol/L for 24 h) synergized with cisplatin to increase the expression of GRP78, GRP94 in HCC cells HepG2, inhibited cancer cell proliferation and induced apoptosis.

Furanocoumarin is one of the main coumarin components of the botanical drug *Hansenia weberbaueriana* (Fedde ex H. Wolff) Pimenov and Kljuykov, which has antiviral effect among others (Zhao J, 2015). Cell experiments (12.5, 25, 50, 100 μmol/L for 24, 48, 72 h) and animal experiments (30 Μm/d for 4 weeks) in mice showed that furanocoumarins inhibit HepJ5 and Mahlavu cell viability and induce apoptosis in HCC cells *in vitro* and *in vivo* by upregulating PERK and CHOP (Huang TY et al., 2023).

Licochalcone B is a chalcone metabolite present in botanical drug *Glycyrrhiza inflata* Batalin, which has antioxidant and various anticancer effects (Chen J et al., 2023). Zhang YY et al. (2022) demonstrated that licoricechalcone B significantly inhibited the viability of HCC cells HepG2 and Huh7 through the Akt/mTOR pathway. This could be attenuated by the PERK inhibitor PD9805 or the JNK inhibitor SP600125.

Saponin fraction from Gleditsia sinensis is a saponin analog present in botanical drug *Gleditsia sinensis* Lam with antioxidant and HCC cell inhibitory effects (Dong et al., 2022). It was found that saponin fraction from Gleditsia sinensis (5, 10, 20 μg/mL for 24 h) dose-dependently upregulated GRP78 and CHOP protein expression in murine HCC cells H22, enhanced the anti-hepatocellular carcinoma effects of the chemotherapeutic drug sorafenib, inhibited HCC cell proliferation and induced apoptosis in HCC cells, which could be attenuated by ERS inhibitor TUDCA (Yin C et al., 2019).

### 4.4 Colorectal cancer

Cucurbitacin, triterpenoid including cucurbitacin B and cucurbitacin E, are triterpenoids from the cucurbitaceae family *Cucumis melo* L., which have therapeutic effects on a wide range of tumors (Hong T et al., 2023; Hsu HL et al., 2023; Yang Y et al., 2023). Huang JL et al. (2023) found that cucurbitacin B (0.005, 0.01, 0.05, 1, 5, 10, 20, 40, 100 μM for 48 h) inhibited the proliferation and induced apoptosis of CRC cells HT-29 and SW620 by concentration-dependently increasing the protein expression of p-PERK, p-eIF2α, ATF4, IRE1α and XBP1. After CHOP knockdown, the apoptosis rate was significantly decreased. Sui HH et al. (2020) reported that cucurbitacin E (0.001, 0.01, 0.1, 1, 10 μmol/L for 24, 48, 72 h) upregulated the expression of CHOP and GRP78 in HT-29 cells and induced apoptosis of CRC cells.

Emodin is an anthraquinone metabolite extracted from a botanical drug *Rheum officinale* Baill with anti-inflammatory and anticancer effects (Lan WB et al., 2023). Liu BR et al. (2016) observed that emodin (30, 60, 90 μmol/L for 48 h) inhibited the proliferation and promoted the apoptosis of CRC cells SW620 through concentration-dependent upregulation of GRP78, CHOP and cleaved Caspase-12 expression. The above effects were attenuated by the addition of the ERS inhibitor 4-PBA, indicating that emodin inhibits CRC via ERS.

Konjac glucomannan, a polysaccharide from botanical drug *Amorphophallus konjac* K. Koch or *Amorphophallus variabilis* Blume sinensis Belval is able to fight against a variety of tumors (Tao QQ et al., 2020). Lu MJ and Chu WJ (2021) observed that the konjac glucomannan (50 μmol/L for 5 d) dose-dependently upregulated the protein levels of cleaved caspase-3, cleaved caspase-9, Bax, PERK, p-eIF2α, ATF4, and CHOP, and downregulated the protein level of Bcl-2, induced apoptosis, and reversed the resistance to 5-Fluorouracil (5-FU) in CRC cells HCT-28.

Bufalin is a steroid presented in the secretion of the botanical drug *Bufo gargarizans* Cantor or *Bufo melanostictus* Schneider, which has good anticancer effects (Zheng YD et al., 2023). Shang J et al. (2023) reported that bufalin (2.5, 5, 10 nmol/L for 48 h) leaded to concentration-dependent upregulation of Bax, GRP78, p-PERK, p-eIF2α, and CHOP proteins expression, downregulation of Bcl-2 and eIF2α, inhibition of CRC cell proliferation, and induction of cancer cell apoptosis in HCT116 cells. The above effects were attenuated by the addition of the ERS inhibitor 4-PBA.

Dehydrodiisoeugenol is a lignan-like metabolite from botanical drug *Myristica fragrans* Houtt with antibacterial, antioxidant and other effects (Xu J et al., 2022). Li C et al. (2023) observed that dehydrodiisoeugenol (10, 20, 30, 40, 50, 60, 70, 80 μM for 24,48,72 h) dose-dependently upregulated BiP, Ero2-Lα, PERK, eIF2α, p-eIF1α, IRE1α, XBP-48s and CHOP expression in HCT116 and SW620 cells, blocking the CRC cell cycle at G1/S, inducing cell autophagy, and inhibiting cell proliferation and tumor growth. The above effects were attenuated after knockdown of PERK or IRE1α, indicating the anti-tumor effects mentioned above are achieved by ERS. Animal study (40 mg/kg/2d for 14 d) also confirmed its specific inhibitory effect on CRC growth in mice.

### 4.5 Other digestive system tumors

Usnic acid, a furan metabolite derived from the botanical drug *Usnea diffracta* Vain, has anti-inflammatory and anti-tumor effects (Xie LS et al., 2023). Gimła M et al. (2023) demonstrated that usnic acid upregulated the expression of BIP, IRE1α and GADD153 in human PDA cells MIA PaCa-2 and PANC-1, blocking the cell cycle at G0/G1, inhibiting tumor growth and inducing apoptosis.

### 4.6 Multiple digestive system tumors

Some extracts, as the studies proved, have treatment effect on more than one digestive system tumor.

Gambogenic acid is a flavonoid isolated from the botanical drug *Garcinia hanburyi* Hook. f and has a good multi-pathway anti-tumor effect but low toxicity (Zhan et al., 2020). Xu T and Li QL (2017) reported that compared with blank control group, gambogenic acid (1, 2, 4 μmol/L for 24 h) affected human nasopharyngeal carcinoma CNE-2Z cells growth and proliferation and induced apoptosis in a concentration-dependent manner by downregulating GRP78 and upregulating CHOP and ATF4 protein expression. Moreover, TT Dai et al. (2014) found that gambogenic acid (2.5, 5, 7.5 μmol/L for 24 h) concentration-dependently inhibited the proliferation and induced apoptosis of CRC cells HCT116, which could be inhibited by the ERS inhibitor 4-PBA and inversely proved to be realized by the ERS pathway.

Osthole, extracted from the fruit of the botanical drug *Cnidium monnieri* (L.) Cuss, is a coumarin analog possessing anti-inflammatory, antioxidant, antifibrotic, and antitumor effects (Wang, XW et al., 2023; Zhao, LN et al., 2023). Pan et al. (2019) found that osthole (20, 40, 60, 80, 100 μM for 24, 48, 72 h) concentration-dependently promoted GRP78, CHOP, ATF-4, GADD34, and XBP-1s expression, downregulated Bax expression, blocked cancer cells at G1 phase, inhibited the proliferation of HCC cells SMMC7721 and induced the apoptosis. In addition, Zhou XH et al. (2021) demonstrated that osthole (25, 50, 100 μM for 24 h) could upregulate GRP78, p-PERK/PERK, p-elF2α/elF2α and CHOP expression, to effectively inhibit the proliferation of HCT-29 and to induce apoptosis in CRC cells, which could be blocked by the autophagy inhibitor 3 MA and ERS inhibitor 4-PBA.

Curcumin is a diketone from the root of botanical drug *Curcuma longa* L with good antibacterial, antioxidant, and antitumor effects (Lei H et al., 2023; Zhang X et al., 2023). Earlier study observed that curcumin (2.5, 5, 10, 20, 50 μM for 12, 24, 48 h) could time- and concentration-dependently promote the expression of GRP78, Caspase-12, and CHOP, inhibit the proliferation of HCC cells SMMC-7721 and induce apoptosis (Xu XM et al., 2018). Li YY and Lu MJ (2020) demonstrated that curcumin (25,50 μmol/L for 1d) could reduce the activity of HCC cells SMMC-7721 and induce apoptosis through ERS signaling pathway. Another study also showed that curcumin (20, 40, 60 μmol/L for 24, 48, 72 h) dose-dependently upregulated the expression of GRP78, p-elF2α, p-JNK, CHOP, and Caspase-4 in BEL-7404 cells, inhibited HCC cell proliferation, and induced apoptosis (Zhang et al., 2021). Huang et al. (2017) reported that curcumin (0.001, 0.01, 0.1, 1, 10 μmol/L for 24,48,72 h) was able to individually or synergized with irinotecan to increase the expression of BIP, PDI, and CHOP in LoVo and HCT-29 cells, blocking the CRC cells at S phase and inducing apoptosis. Apoptosis was alleviated after interfering with the CHOP gene. Zhu C et al. (2022) separately demonstrated a similar effect of curcumin on CRC cells CT-26.

Oridonin, a diterpenoid isolated from *Rabdosia rubescens* (Hemsl.) Hara, is able to fight against a variety of tumors such as leukemia and ovarian (He J et al., 2023; Li M and Wang XX, 2023). Zhou F et al. (2023) demonstrated that oridonin (5, 10, 15, 20, 25, 30 μM for 24, 48, 72 h) increased ATF4 and CHOP concentrations of CRC cells NCM460, HCT116, LoVo, SW480, RKO, HeLa, PC-3 and A594, inhibited TCF4 activation and induced ERS dysregulation and death of tumor cells. Animal study demonstrated the inhibitory effect of oridonin (160 mg/kg/d for 14 d) on the proliferation of TCF4 and Mock cells as well. What’s more, Liu DL et al. (2022) observed that compared to the control, oridonin (5, 10, 20, 40, 80 μmol/L for 24, 48, 72 h) inhibited the proliferation and induced apoptosis of PDA SW1990 and Panc-1 cells as well in a time- and dose-dependent manner by upregulating the protein expression levels of GRP78, CHOP, and cleaved Caspase-12, which could be blocked by the ERS blocker 4-PBA.

Piperlongumine is an alkaloid from botanical drug *Piper nigrum* L. with anti-inflammatory and anti-tumor effects (Li YD et al., 2022). Zou P et al. (2016) found that piperlongumine (0.625, 1.25, 2.5, 5, 10, 20 μmol/L for 24, 48 h) upregulated the expression of p-eIF2α, ATF4, and CHOP in SGC-7901 and BGC-823 cells, activating the lethal ERS to promote GC cell apoptosis. Upon inhibition of CHOP, apoptosis was attenuated. Animal experiment also confirmed the inhibitory effect of piperlongumine (4.12 mg/kg/2d for 15d) on GC proliferation in mice. A recent study observed that piperlongumine also synergized with a chemotherapeutic agent bortezomib to inhibit cholangiocarcinoma (Naradun N et al., 2023).

## 5 Conclusion

Traditional Chinese medicine theory classifies the development of diseases into two major causes: “Zhengqi deficiency (insufficient regulation of the human body)" and “Xie qi sufficiency (interference of external pathogenic elements)", which in the development of digestive system tumors is specifically manifested as the accumulation of various abnormal metabolites and damages exceeding the critical point under the action of pathogenic factors, leading to cancerous transformation. In this process, the modification of ER and adjustment of functional proteins throughout the body is an important manifestation of the self-regulatory function of Zhengqi, and its dysfunction (ERS) will accelerate the progression of the tumors.

With the development of molecular biotechnology in recent years, the analysis and extraction of active metabolites in Chinese botanical drugs have provided more efficient options for basic research and clinical application. The above studies have proved that many Chinese botanical drug extracts can enhance the antitumor effects of ERS and have great potential for application in the increasingly prevalent digestive system tumors due to their rich variety, stable sources, clear targets, low toxicity, and remarkable effects. By linking the frontier pathological findings of digestive system tumors with known active metabolites of Chinese botanical drug, we can shorten the transformation period of the former and broaden the clinical application of the latter, which can contribute significantly to the development of antitumor drugs. However, on the one hand, there is still a lack of sufficient research on the cooperative use of Chinese botanical drug extracts, which makes it difficult to utilize the advantages of multi-targets and multi-pathways of Chinese botanical drugs. On the other hand, most of the studies are based on cellular experiments, and the pharmacokinetic process of drug metabolism is not consistent with the real world, which adds certain difficulties to the clinical translation of the research results. Therefore, in addition to focusing on the process of ERS action, future research should also pay more attention to the following points: (1) using validated clinical prescriptions of Chinese botanical drugs as reference to further probe the synergistic application of their extracts; (2) conducting more animal experiments to further study the metabolism process of the drug extracts *in vivo*; (3) exploring the combing use with modern medicines to improve the cost-effect ratio and accelerate the translation of the research results into clinical therapeutic regimens.

In summary, a large body of evidence imply that Chinese botanical drug extracts modulating the ERS pathway and thus treating digestive tumors is a viable medical perspective, which still has a huge potential waiting to be explored.

## Abbreviations

4-PBA, 4-phenyl butyric acid; 5-Fu, 5-fluorouracil; ASK1, apoptotic signaling kinase; ATF4, activating transcription factor 4; ATF6, activating transcription factor 6; Bcl-2, B-cell lymphoma-2; CHOP, CCAAT/enhancer binding protein homologous protein; CRC, colorectal cancer; DMSO, Dimethyl sulfoxide; EAC, esophageal adenocarcinoma; EC, esophageal cancer; ESSC, esophageal squamous cell carcinoma; eIF2α, eukaryotic translation initiation factor 2α; ERAD, endoplasmic reticulum-associated degradation; ERS, endoplasmic reticulum stress; ESCC, esophageal squamous cell carcinoma; GADD, growth arrest and DNA damage-inducible protein; GC, gastric cancer; GRP78, glucose regulated protein; HCC, hepatocellular carcinoma; IRE1, inositol-requiring enzyme 1; JNK, c-Jun N-terminal kinase 1; Nrf-2, nuclear factor-erythroid 2-related factor-2; PDA, pancreatic cancer; PERK, protein kinase RNA-like endoplasmic reticulum kinase; RIDD, regulated IRE1α-dependent decay; TLR4, toll-like receptor; TRAF2, tumor necrosis factor receptor associated factors 2; UPR, unfolded protein response; XBP1, X-box binding protein 1.
